# The Fabrication of a La_2_Sn_2_O_7_/*f*-HNT Composite for Non-Enzymatic Electrochemical Detection of 3-Nitro-l-tyrosine in Biological Samples

**DOI:** 10.3390/bios13070722

**Published:** 2023-07-10

**Authors:** Balasubramanian Sriram, Sakthivel Kogularasu, Sea-Fue Wang, Guo-Ping Chang-Chien

**Affiliations:** 1 Department of Materials and Mineral Resources Engineering, National Taipei University of Technology, Taipei 106, Taiwan; sricamaro@ntut.edu.tw; 2 Department of Photonics, National Cheng Kung University, Tainan 701, Taiwan; kogularasu@gs.ncku.edu.tw; 3 Institute of Environmental Toxin and Emerging-Contaminant Research, Cheng Shiu University, Kaohsiung 833301, Taiwan; 4 Super Micro Mass Research and Technology Center, Cheng Shiu University, Kaohsiung 833301, Taiwan; 5 Center for Environmental Toxin and Emerging-Contaminant Research, Cheng Shiu University, Kaohsiung 833301, Taiwan

**Keywords:** La_2_Sn_2_O_7_, f-HNT, 3-nitro-l-tyrosine, electrochemical biosensor, real-time sensing

## Abstract

Reactive oxygen and nitrogen species (RONS), including 3-nitro-l-tyrosine, play a dual role in human health, inducing oxidative damage and regulating cellular functions. Early and accurate detection of such molecules, such as L-tyrosine in urine, can serve as critical biomarkers for various cancers. In this study, we aimed to enhance the electrochemical detection of these molecules through the synthesis of La_2_Sn_2_O_7_/f-HNT nanocomposites via a simple hydrothermal method. Detailed structural and morphological characterizations confirmed successful synthesis, consistent with our expected outcomes. The synthesized nanocomposites were utilized as nanocatalysts in electrochemical sensors, showing a notable limit of the detection of 0.012 µM for the real-time detection of 3-nitro-l-tyrosine. These findings underscore the potential of nanomaterial-based sensors in advancing early disease detection with high sensitivity, furthering our understanding of cellular oxidative processes.

## 1. Introduction

Early identification and screening of high-risk populations are effective approaches for lowering cancer incidence [1,2]. Cancer biomarkers present in biofluids such as urine, serum, and saliva are crucial in detecting and screening various cancers in humans [3,4]. Early detection and screening can potentially enhance treatment options and decrease cancer mortality rates. Previous studies have identified L-tyrosine in urine as a significant biomarker for certain types of cancers [5]. Overproduction of reactive nitrogen and oxygen species creates an imbalance that causes free radicals, including peroxynitrite radicals, nitryl chloride, nitrogen dioxide, and nitrous acid, followed by nitrosative/oxidative stress [6,7]. These free radicals can alter the aromatic amino acids, such as tyrosine, due to their hydrophilic nature. The ortho position of the phenolic ring in tyrosine experiences nitration in the existence of ONOO free radicals, leading to the formation of 3-nitro-l-tyrosine [7]. Excessively high levels of 3-nitro-l-tyrosine serve as in vivo biomarkers for nitrosative stress and are repeatedly associated with numerous chronic illnesses, as well as Alzheimer’s, neurodegenerative, and neuropsychiatric diseases [8]. Therefore, the identification and quantification of 3-nitro-l-tyrosine in biological trials can provide valuable insight into the presence and severity of nitrosative stress-related diseases [9]. Currently, low-cost paper-based devices combined with solid-phase extraction (SPE) technology are widely used in various fields such as biomedical, environmental, and food monitoring due to their simplified analytical systems. However, the integration of paper as a material platform for the development of sensitive and selective electrochemical detection has been limited to only a few studies [10]. For instance, a fully printed SPE was developed to directly target 3-nitrotyrosine through its oxidation, with a maximum peak observed at approximately 0.75 V. However, this direct reading approach may result in inaccurate data when analyzing complex samples [11]. This is because every compound present in the sample that can oxidize at a lower potential can contribute to a positive error. Therefore, there is a need to develop more accurate and selective electrochemical detection methods that can be integrated with paper-based devices to improve their reliability and sensitivity for the analysis of complex samples [12,13,14,15].

Pyrochlore metal oxides, which have a general formula of A_2_B_2_O_7_, are an important type of functional material [16,17]. They are considered promising electrocatalysts in the sensors due to their physicochemical properties, numerous oxygen vacancies, and adaptable structure [18]. Recent research has revealed that pyrochlore stannate’s surface contains plentiful oxygen species, which facilitate the effective movement of oxygen ions. This characteristic makes pyrochlore stannate, when properly organized and structurally stable, an excellent choice for modifying electrodes. Among the diverse lanthanide stannates, La_2_Sn_2_O_7_ stands out as a highly desirable electrode option [17,19]. This is due to the high spin–orbital coupling interaction present in this compound, resulting in a unique crystal structure.

Halloysite nanotubes (HNTs) possess favorable surface chemistry and are a type of natural nano clay found in deposits worldwide, with the largest deposits located in New Zealand and Utah (U.S.) [20]. Due to their lower toxicity, they can be considered a safer nanomaterial. The specific geological origin of halloysite nanotubes affects their purity [21]. These nanotubes have a hollow tubular figure with aluminosilicate sheets that have a spiral-like morphology, portrayed by a micrometer-scale length with external diameters ranging from 60 to 300 nm, and internal diameters of 10 to 60 nm. The interlayer distance depends on the hydration state of the halloysite and is either 1 or 0.6 nm. Its unitary cell formula is similar to the common kaolinite, Al_2_Si_2_O_5_(OH)_4_·nH_2_O, excluding the existence of H_2_O molecules (typically 2) that are located between the adjoining clay layers [22]. HNTs possess unique properties that distinguish them from other clay minerals and kaolin. One of these properties is the existence of an empty lumen within their structure, which makes them an ideal substrate for loading different types of molecules [23]. Furthermore, the exterior surface of HNTs facilitates the adsorption of a wide range of organic compounds. The surface of HNTs also contains reactive functional groups that enable them to cooperate with other molecules through various mechanisms [24].

HNTs exhibit compelling characteristics such as thermal stability, controlled surface chemistry, and cost-effectiveness due to their hollow shape. These unique properties make HNTs an attractive material for various advanced fields [25,26]. For example, they can be utilized to create new nanocomposites that can electrochemically sense different analytes, like hydrogen peroxide and glucose. Moreover, due to their material chemistry, HNTs are suitable for catalyzing chemical and biochemical reactions. The surface chemistry of HNTs is also easy to control chemically, and their hydroxyl groups facilitate functionalization to enhance their properties [27]. Our research group has recently explored the electrochemical sensing abilities of La_2_Sn_2_O_7_ nanoparticles [28]. After successful outcomes, the La_2_Sn_2_O_7_ nanoparticles are upgraded to nanocomposites with the help of HNTs [17]. Due to its synergetic effect, the properties of the La_2_Sn_2_O_7_ nanoparticles are enhanced enough for an excellent electrochemical sensor.

As previously mentioned, morphological, spectroscopic, and voltammetric experiments were used to explore the electrocatalytic activity of La_2_Sn_2_O_7_/*f*-HNT nanocomposites, which were sustainably prepared by the hydrothermal method. The prepared La_2_Sn_2_O_7_/*f*-HNT nanocomposites were used to detect 3-nitro-l-tyrosine at extremely low levels in real time after its composition was confirmed.

## 2. Experimental Section

### 2.1. Materials

The necessary chemicals, namely lanthanum(III) nitrate hexahydrate, tin(II) chloride, cetyltrimethylammonium bromide (CTAB), and sodium hypophosphite (NaH_2_PO_2_), were acquired from Sigma Aldrich without requiring additional purification, ensuring their quality and reliability. The screen-printed carbon electrodes (SPCEs) utilized in the study were obtained from Zensor.Pvt.Ltd, a reputable supplier based in Taiwan, which is known for providing reliable and high-performance electrodes. To facilitate the electrochemical investigations, a 0.1 M phosphate buffer (PB) was used as the assisting electrolyte, chosen for its ability to maintain a stable pH and create an appropriate ionic environment for the electrochemical reactions under study. These carefully selected chemicals and materials, along with the high-quality commercial electrodes and optimized electrolytes, contributed to the establishment of a robust experimental setup, ensuring the accuracy and reproducibility of the electrochemical studies conducted. We have made provisions for a more thorough understanding of our research by supplementing our study with extensive details on both the instrumentation employed and the methodology followed for sample preparation. These details, which are critical to our research, can be found in the the Supplementary Materials.

### 2.2. Synthesis of La_2_Sn_2_O_7_ Nanoparticles

**Scheme 1:**
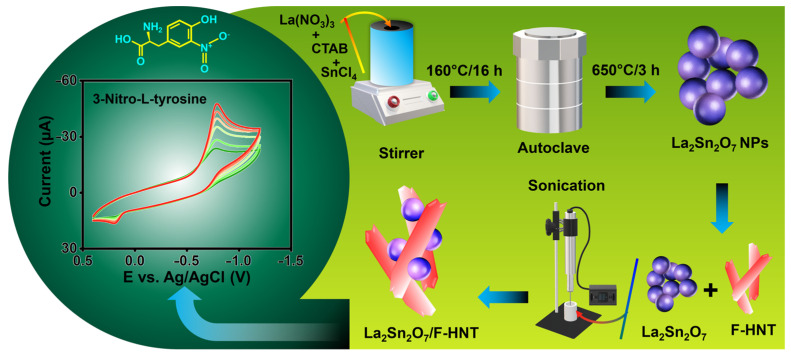
A schematic illustration of the synthesis of La_2_Sn_2_O_7_ and La_2_Sn_2_O_7_/*f*-HNT for the electrochemical detection of 3-nitro–L–tyrosine.

In this study, La_2_Sn_2_O_7_ nanoparticles were synthesized through a straightforward hydrothermal method, as depicted in Scheme 1. To initiate the synthesis, a solution containing lanthanum(III) nitrate hexahydrate (0.5 M) and tin(II) chloride (0.5 M) in distilled water was prepared and continuously stirred. In addition, CTAB was incorporated into the solution and mixed at room temperature for a duration of 2 h. Subsequently, the homogeneous solution was transferred into a Teflon-lined autoclave and maintained at a temperature of 160 °C for a period of 16 h. Once the reaction was completed, the autoclave was allowed to cool to room temperature. The resulting product underwent multiple rounds of centrifugation, using water and ethanol, followed by drying in a hot air oven at 80 °C for 24 h. Finally, the LaSn(OH)_6_ was subjected to calcination at 650 °C for 3 h, leading to the successful formation of La_2_Sn_2_O_7_ nanoparticles.

### 2.3. Synthesis of f-HNT

The synthesis of functionalized halloysite nanotubes (*f*-HNTs) in this study followed a previously established procedure [25]. To initiate the synthesis, 0.5 g of pure HNTs were carefully mixed with 50 mL of 0.5 M H_2_SO_4_ and subjected to continuous stirring at a controlled temperature of 60 °C for a duration of 5 h. During this period, the reaction proceeded, resulting in the desired functionalization of the HNTs. Once the reaction was completed, the resulting product was separated from the reaction mixture through centrifugation at 6000 revolutions per minute (rpm) for 5 min. This centrifugation step allowed for the separation of the *f*-HNTs from the residual solution. Subsequently, the obtained solid product was subjected to thorough washing using a mixture of ethanol and water, which was repeated several times to ensure the removal of any residual impurities and to reach a neutral pH. To achieve a neutral pH, the washing process involved multiple rinses with the ethanol/water mixture and was carefully performed to eliminate any traces of acidic or basic substances. By carefully controlling the pH, the stability and compatibility of the *f*-HNTs in subsequent applications were ensured. Finally, after the extensive washing process, the purified f-HNTs were dried in a controlled environment at a temperature of 50 °C for a duration of 24 h. This drying process allowed for the complete removal of any remaining moisture and solvent, resulting in the obtainment of dry, stable *f*-HNTs that were ready for further utilization in the subsequent stages of the research.

### 2.4. Synthesis of La_2_Sn_2_O_7_/f-HNT

To prepare the La_2_Sn_2_O_7_/*f*-HNT nanocomposite as an effective electrode modifier for electrochemical testing, we implemented a meticulous approach involving the optimization of the weight ratio between *f*-HNT and La_2_Sn_2_O_7_. By carefully determining the appropriate ratio, we aimed to achieve a synergistic combination of the two components, maximizing their potential benefits in enhancing the electrochemical performance of the electrode. To initiate the preparation process, the calculated amounts of *f*-HNT and La_2_Sn_2_O_7_ were combined and mixed with distilled water. To ensure the formation of a homogeneous and well-dispersed nanocomposite, the mixture underwent 30 min of ultrasonication. Ultrasonication is a common technique used to break up agglomerations and promote the dispersion of nanoparticles, enabling better interaction between the components and enhancing the overall homogeneity of the resulting composite material. The ultrasonication process provided mechanical energy that facilitated the dispersion of *f*-HNT and La_2_Sn_2_O_7_, allowing for the formation of a uniform solution. The interaction between the nanoparticles was promoted, ensuring their thorough integration and distribution within the composite matrix. This step was crucial to enhance the accessibility of active sites and optimize the electrochemical properties of the modified electrode. Following the formation of the La_2_Sn_2_O_7_/*f*-HNT nanocomposite, it was employed as an electrode modifier in subsequent electrochemical examinations. By incorporating the nanocomposite onto the electrode surface, it aimed to improve the electrochemical performance, such as enhancing the electron transfer kinetics, increasing the specific surface area, and facilitating the adsorption and detection of target analytes. The use of the nanocomposite as an electrode modifier provided an opportunity to leverage the unique properties of both *f*-HNT and La_2_Sn_2_O_7_, creating a synergistic effect that could potentially lead to superior electrochemical performance compared to individual components or unmodified electrodes.

### 2.5. Fabrication of Modified Electrodes

To prepare the SPCE for electrochemical experiments, a thorough cleaning process was employed to ensure the removal of any contaminants on the reactive surface. Initially, 0.5 mg of alumina slurry was applied to the electrode, and ultrasonication was carried out for a duration of 10 min. This step helped to dislodge and remove any impurities present on the electrode surface. Following sonication, the electrode was immersed in ethanol to further cleanse the surface and eliminate any residual alumina particles or other contaminants. To complete the cleaning procedure, the electrode underwent a cycling process between −0.8 and 0.8 V in a phosphate buffer (PB) with a pH of 7. This cycling was performed continuously for 25 cycles, effectively removing any remaining adsorbed species and ensuring the establishment of a clean, bare electrode surface.

Next, the La_2_Sn_2_O_7_/*f*-HNT nanocomposite was prepared for deposition onto the SPCE surface. A suspension containing 6 mg/mL of La_2_Sn_2_O_7_/*f*-HNT dispersed in 1 mL of ethanol was sonicated for 20 min. This sonication step aimed to achieve a homogeneous and well-dispersed nanocomposite suspension, enabling uniform deposition on the electrode surface. The prepared La_2_Sn_2_O_7_/f-HNT suspension was then applied to the SPCE surface in a controlled amount of 8 µL. Care was taken to ensure precise and consistent application to obtain an even and optimized coverage of the electrode surface. To facilitate the drying process, the electrode was baked at a temperature of 50 °C, allowing the solvent (ethanol) to evaporate while promoting the adhesion of the La_2_Sn_2_O_7_/*f*-HNT nanocomposite to the electrode surface. Upon completion of these steps, the La_2_Sn_2_O_7_/*f*-HNT/SPCE electrode was fully prepared for subsequent experiments. The modified electrode, with its tailored nanocomposite layer, was anticipated to exhibit enhanced electrochemical properties, such as improved electron transfer kinetics, enlarged active surface area, and enhanced analyte adsorption capabilities. The meticulously conducted cleaning procedure, nanocomposite suspension preparation, and controlled deposition onto the electrode surface all played vital roles in the successful preparation of the La_2_Sn_2_O_7_/*f*-HNT/SPCE for use in subsequent electrochemical investigations.

### 2.6. Real Samples Preparation

To perform the spiked detection analysis, urine and saliva samples were collected from a healthy volunteer. The samples were first subjected to ultrafiltration, a process that separates the smaller molecules and particles from the larger ones by applying pressure. This step ensured the removal of any unwanted substances and debris present in the samples. Notably, the ultrafiltration process was performed without precipitation, which could introduce additional contaminants or affect the integrity of the samples. Following ultrafiltration, the resulting filtrates were diluted in a phosphate buffer (PB) with a pH of 7. This step aimed to create a standardized and suitable environment for the subsequent analysis. The PB solution, with its defined pH level, provided a stable and consistent background for the spiked detection analysis, enabling accurate and reliable measurements. The prepared solutions, obtained from the ultrafiltrated and diluted urine and saliva samples, were then utilized as real samples for the spiked detection analysis. Spiking involves the addition of known amounts of target analytes or substances of interest to the samples to evaluate their detectability and measure their concentration levels accurately. By spiking the prepared solutions, the researchers could assess the sensitivity and efficacy of the detection method used, ensuring its suitability for the analysis of real-life samples.

## 3. Results and Discussion

### 3.1. Characterization of f-HNT, La_2_Sn_2_O_7_, and La_2_Sn_2_O_7_/F-HNT

#### 3.1.1. X-ray Diffraction (XRD)

**Figure 1:**
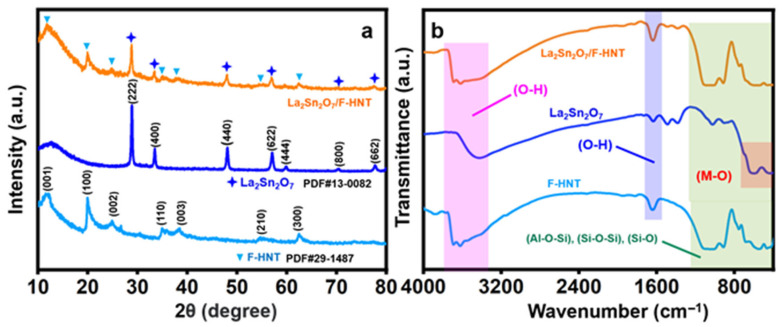
(**a**) XRD pattern and (**b**) FT-IR spectrum of *f*-HNT, La_2_Sn_2_O_7_, and La_2_Sn_2_O_7_/*f*-HNT.

In Figure 1a, the well-defined diffraction patterns obtained correspond to the cubic crystalline structure, with Miller indices of (2 2 2), (4 0 0), (4 4 0), (6 2 2), (4 4 4), (8 0 0), and (6 6 2) [17,28]. The interpretation of the space group (Fd-3m) and cell parameters (a = b = c = 10.70) supports the formation of La_2_Sn_2_O_7_ nanoparticles (star shape), which is also consistent with our recent research work. However, the addition of *f*-HNT gave rise to several peaks. The XRD pattern of the synthesized *f*-HNT (triangle shape) demonstrates the degree of purity and quality of crystallization of the *f*-HNT being studied. The findings indicate a complete match between all diffraction peaks and *f*-HNT, affirming the presence of *f*-HNT based on the indexed crystal planes of (001), (100), (002), (110), (003), (210), and (300) at diffraction angles [25].

#### 3.1.2. Fourier-Transform Infrared Spectroscopy (FT-IR)

The distinct peaks observed in the Fourier-transform infrared (FT-IR) spectrum of *f*-HNT provide valuable insights into the molecular structure and bonding characteristics of the material. Specifically, the peak observed at around 1043 cm^−1^ can be attributed to the stretching vibration of Si–O bonds within the plane of the *f*-HNT structure. Furthermore, the peak observed at approximately 1634 cm^−1^ indicates the deformation of water molecules present in the *f*-HNT. Additionally, there are corresponding peaks observed at 688 cm^−1^, 534 cm^−1^, and 467 cm^−1^, which can be associated with the stretching vibration of Si–O bonds, the vibration of Al–O–Si bonds, and the deformation of Si–O–Si bonds, respectively. These peaks further elucidate the bonding environment within the *f*-HNT structure. An accompanying image provides visual representations of the molecular vibrations occurring within *f*-HNT. It depicts the deformation vibration of the internal hydroxyl group and the in-plane stretching of Si–O–Si and O–H molecules. The specific frequencies associated with these vibrations are observed at 999.8 cm^−1^, 910 cm^−1^, and 752 cm^−1^, respectively. These vibrational modes are key indicators of the structural and chemical properties of the *f*-HNT material. In the case of La_2_Sn_2_O_7_ nanoparticles, the FT-IR spectrum reveals distinct peaks that correspond to the stretching vibrations of the constituent ions within the crystal matrix. Notably, a prominent peak observed at 408 cm^−1^ can be attributed to the strong La–O stretching vibration. Similarly, a peak at 599 cm^−1^ confirms the presence of Sn–O stretching vibration within the SnO6 octahedron structure. Additionally, two other peaks at 3413 cm^−1^ (broad) and 1631 cm^−1^ (weak) are identified as the bending and stretching vibrations of OH functionalities. It should be noted that these OH groups are a result of the physical adsorption of H_2_O molecules during the sample preparation process for FT-IR measurements and are significantly influenced by hydrogen bonding. Another small peak at 1058 cm^−1^ corresponds to the strong vibration of the carbonate functional group, likely due to contact with the ambient atmosphere. The position and intensity of this carbonate peak may slightly vary depending on the lanthanum content. Collectively, these observations indicate the presence of a physical interface within the composite material, highlighting the intricate molecular interactions occurring within the La_2_Sn_2_O_7_/*f*-HNT nanocomposite. [25,28]

#### 3.1.3. Microscopic Studies

**Figure 2:**
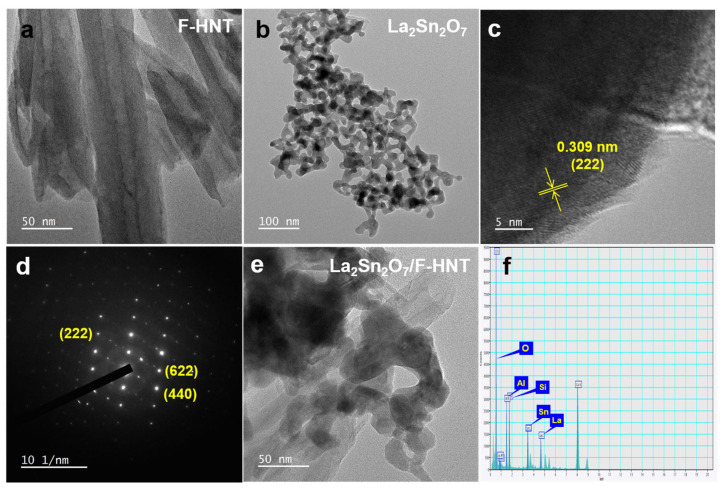
TEM images of *f*-HNT (**a**), La_2_Sn_2_O_7_ (**b**), lattice fringes (**c**), and SAED patterns (**d**) at different magnifications. (**e**) TEM image and (**f**) EDX analysis of La_2_Sn_2_O_7_/*f*-HNT.

To gain a comprehensive understanding of the morphologies of *f*-HNT, La_2_Sn_2_O_7_, and the La_2_Sn_2_O_7_/*f*-HNT nanocomposite, a detailed examination of typical TEM images was conducted. In Figure 2a, the TEM images clearly depict the hollow structure of *f*-HNTs, with an average diameter ranging from 20 to 50 nm. This unique one-dimensional characteristic offers several advantages in terms of enhancing electrochemical performance. Notably, it provides a large number of active sites and facilitates efficient electronic conductivity, contributing to improved overall performance. Moving on to La_2_Sn_2_O_7_, Figure 2b reveals high-resolution TEM (HR-TEM) images that confirm its nanocrystalline nature. The average particle size of La_2_Sn_2_O_7_ is estimated to be around 10 to 15 nm. The well-defined lattice fringes shown in Figure 2c and the corresponding selected area electron diffraction (SAED) pattern in Figure 2d further support the good crystallinity of La_2_Sn_2_O_7_. The higher magnification image in Figure 2d provides a closer look at the lattice fringes, with a calculated d-spacing value of 0.311 nm, corresponding to the interplanar spacing of the XRD plane of 222. The SAED pattern validates the highly crystalline nature of La_2_Sn_2_O_7_, as the bright spots align with the diffraction planes specific to La_2_Sn_2_O_7_, which is consistent with previous investigations. Now, turning our attention to the La_2_Sn_2_O_7_/*f*-HNT composite, Figure 2e presents HR-TEM images that clearly demonstrate the anchoring of numerous La_2_Sn_2_O_7_ nanoparticles onto the surface of *f*-HNT. This composite, known as La_2_Sn_2_O_7_/*f*-HNT, exhibits several advantages for electrochemical processes, including a significantly enlarged active surface area and efficient electronic transportation. The TEM images successfully showcase the successful integration of the two-component system, highlighting the preservation of their distinct and efficient individual features without any detrimental effects during the composite formation. Moreover, an energy-dispersive X-ray spectroscopy (EDX) analysis of the La_2_Sn_2_O_7_/*f*-HNT composite was performed, confirming the uniform distribution of each element (La, Sn, O, Al, and Si) without the presence of additional impurities. This is illustrated in Figure 2f, where the EDX spectrum indicates the reliable incorporation of each element within the composite material. By closely examining the TEM images and conducting EDX analysis, a comprehensive understanding of the morphologies and elemental distribution in *f*-HNT, La_2_Sn_2_O_7_, and the La_2_Sn_2_O_7_/*f*-HNT nanocomposite is achieved. These findings emphasize the favorable characteristics and successful integration of the individual components, paving the way for enhanced electrochemical performance in various applications.

### 3.2. Electrochemical Behavior of La_2_Sn_2_O_7_/F-HNT

#### 3.2.1. Electrochemical Detection of 3-Nitro-l-tyrosine

**Figure 3:**
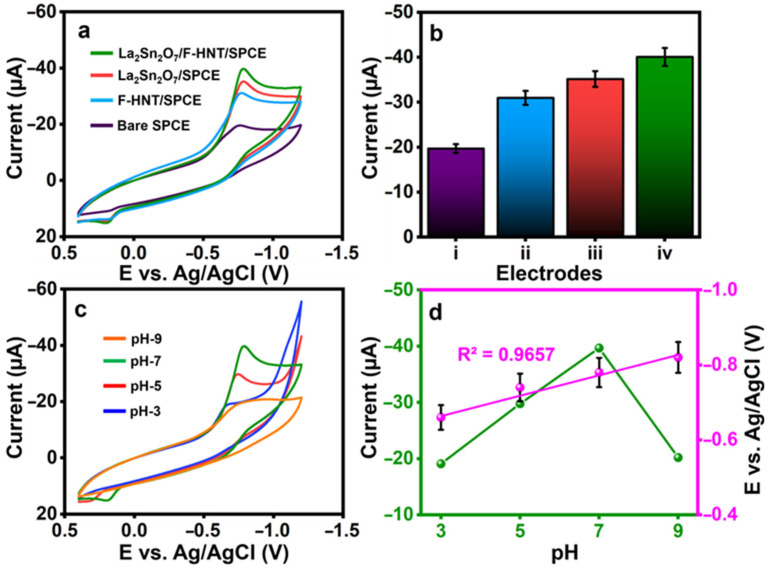
(**a**) CVs were recorded for the electrodes prepared using 100 μM 3-nitro-l-tyrosine in 0.1 M PB, and (**b**) the current response was graphically represented for specific electrodes (i: bare electrode; ii: *f*-HNT/SPCE; iii: La_2_Sn_2_O_7_/SPCE; iv: La_2_Sn_2_O_7_/*f*-HNT/SPCE). (**c**) CVs were obtained for the electrodes at different pH values, (**d**) the current and potentials were plotted against the pH values, and the pH was adjusted to contain 100 μM 3-nitro-l-tyrosine at La_2_Sn_2_O_7_/*f*-HNT.

The constructed electrode’s cyclic voltammetry curves for the detection of 3-nitro-l-tyrosine were tested in (0.1 M) pH-7. A lower current of I_pc_= −19.7 µA with a reduction peak potential of E_pc_= −0.758 V was observed at a bare SPCE on the detection of 3-nitro-l-tyrosine, as shown in Figure 3a. With peak potentials of E_pc_= −0.769 V and E_pc_= −0.787 V for 3-nitro-l-tyrosine, the *f*-HNT and La_2_Sn_2_O_7_ exhibit better cathodic currents of I_pc_= −30.92 µA and I_pc_= −35.18 µA, respectively. At La_2_Sn_2_O_7_/*f*-HNT, the greatest reduction peak current of I_pc_= −40.07 µA at E_pc_= −0.787 V for the detection of 3-nitro-l-tyrosine was observed. This is because La_2_Sn_2_O_7_ and *f*-HNT have a synergistic electrocatalytic effect with their respective unique properties. Figure 3b shows a visual plot of the obtained results from CV curves. Then, using CV, the impacts of various pH ranges from 3 to 9 are examined. The CVs were collected using the ideal setup and a buffer pH containing 3-nitro-l-tyrosine (100 µM), as shown in Figure 3c. Maximum reduction current response is found at pH 7 when pH is adjusted from 3.0 to 7.0. There is a decrease in current signals after pH 7 is attained. From the results, pH 7 was used for the electrochemical reduction in 3-nitro-l-tyrosine. Figure 3d demonstrates the current, and potentials were plotted against the pH values.

**Figure 4:**
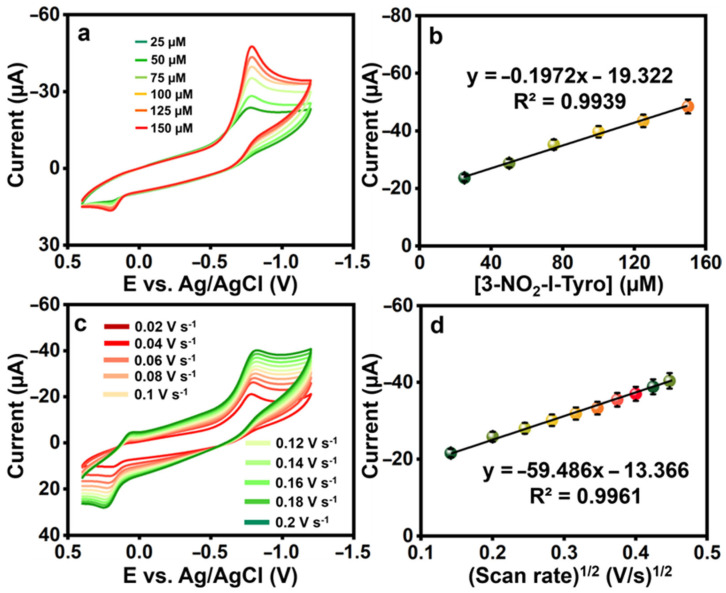
(**a**) The CVs were recorded for various concentrations of 3-nitro-l-tyrosine (25 μM each) at La_2_Sn_2_O_7_/*f*-HNT. (**b**) A calibration plot was generated by plotting the concentration against the current. (**c**) CVs of La_2_Sn_2_O_7_/*f*-HNT containing 25 μM of 3-nitro-l-tyrosine at different scan rates (0.02–0.2 Vs^−1^) in 0.1 M pH-7. (**d**) Calibration plot scan rate (Vs^−1^)^1/2^ vs. peak currents (μA).

To examine the electrocatalytic activity of the La_2_Sn_2_O_7_/*f*-HNT, CVs were produced for the changing addition of 3-nitro-l-tyrosine in 0.1 M pH-7. Figure 4a,b show that the redox peak current response increased linearly when 3-nitro-l-tyrosine concentration ranges increased from 25 to 150 µM, demonstrating the catalytic activity of the La_2_Sn_2_O_7_/*f*-HNT, along with the regression and correlation coefficient equations: (Cathodic) y=−0.1972x−19.322; R²=0.9939.

**Scheme 2:**
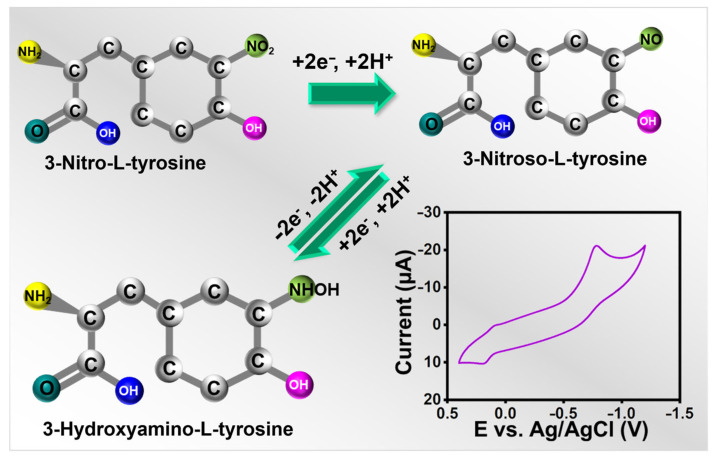
A schematic diagram of the detection mechanism of 3-nitro-l-tyrosine.

Electrochemical sensors must have scan rates since they help determine the kinetics of any analytes. For alternate scans, the CVs were, therefore, recorded at 0.02 to 0.2 V s^−1^ in the presence of 25 µM 3-nitro-l-tyrosine (pH-7), and the results were shown in Figure 4c,d. When the scan rate was raised during the experiment, the redox peak current of 3-nitro-l-tyrosine gradually increased. The possible electrochemical detection mechanism of 3-nitro-l-tyrosine is represented in Scheme 2. Further, this is a case in which the reactions at La_2_Sn_2_O_7_/*f*-HNT/SPCE are controlled by diffusion. The regression and correlation coefficient equations for the square root of the scan rate versus currents are as follows: (Cathodic) y=−0.59486x−13.366; R²=0.9961.

#### 3.2.2. DPV Analysis of 3-Nitro-l-tyrosine at La_2_Sn_2_O_7_/f-HNT

**Figure 5:**
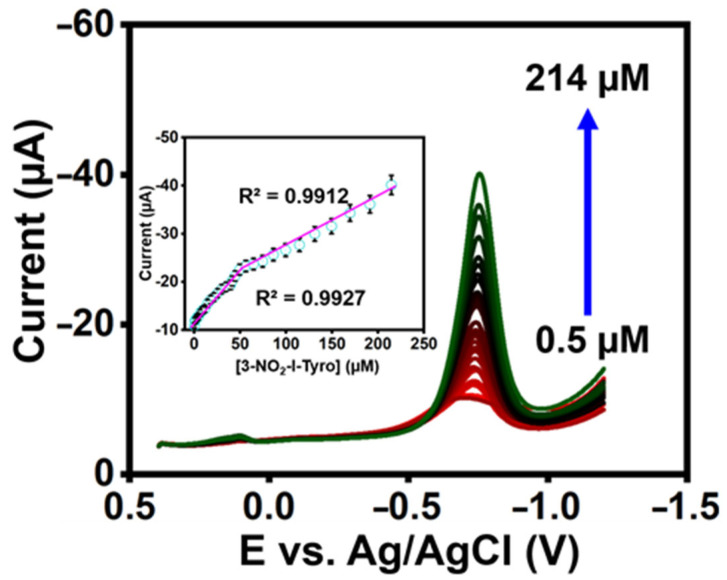
DPV response at La_2_Sn_2_O_7_/*f*-HNT/SPCE by adding 3-nitro-l-tyrosine in PB (0.1 M) (**inset**). Calibration plot of DPV response.

The constructed sensor’s qualitative analysis was investigated using differential pulse voltammetry (DPV). The DPV reaction of 3-nitro-l-tyrosine at La_2_Sn_2_O_7_/*f*-HNT/SPCE in 0.1 M pH-7 is shown in Figure 5. In addition, the reduction peak currents increased with a correlation coefficient of 3-nitro-l-tyrosine R^2^= 0.9927 and R^2^ = 0.9912 as the concentration of 3-nitro-l-tyrosine increased in the range of 0.5–214 µM (Figure 5 inset). Additionally, it was determined that the LOD of the constructed sensor was 0.012 µM for 3-nitro-l-tyrosine (LOD calculation: LOD = 3σ/S) [29,30,31,32,33,34,35,36,37]. The constructed sensor’s outcomes are better than recently published sensors (Table S1).

#### 3.2.3. Reproducibility and Cycle Stability of 3-Nitro-l-tyrosine at La_2_Sn_2_O_7_/f-HNT/SPCE

**Figure 6:**
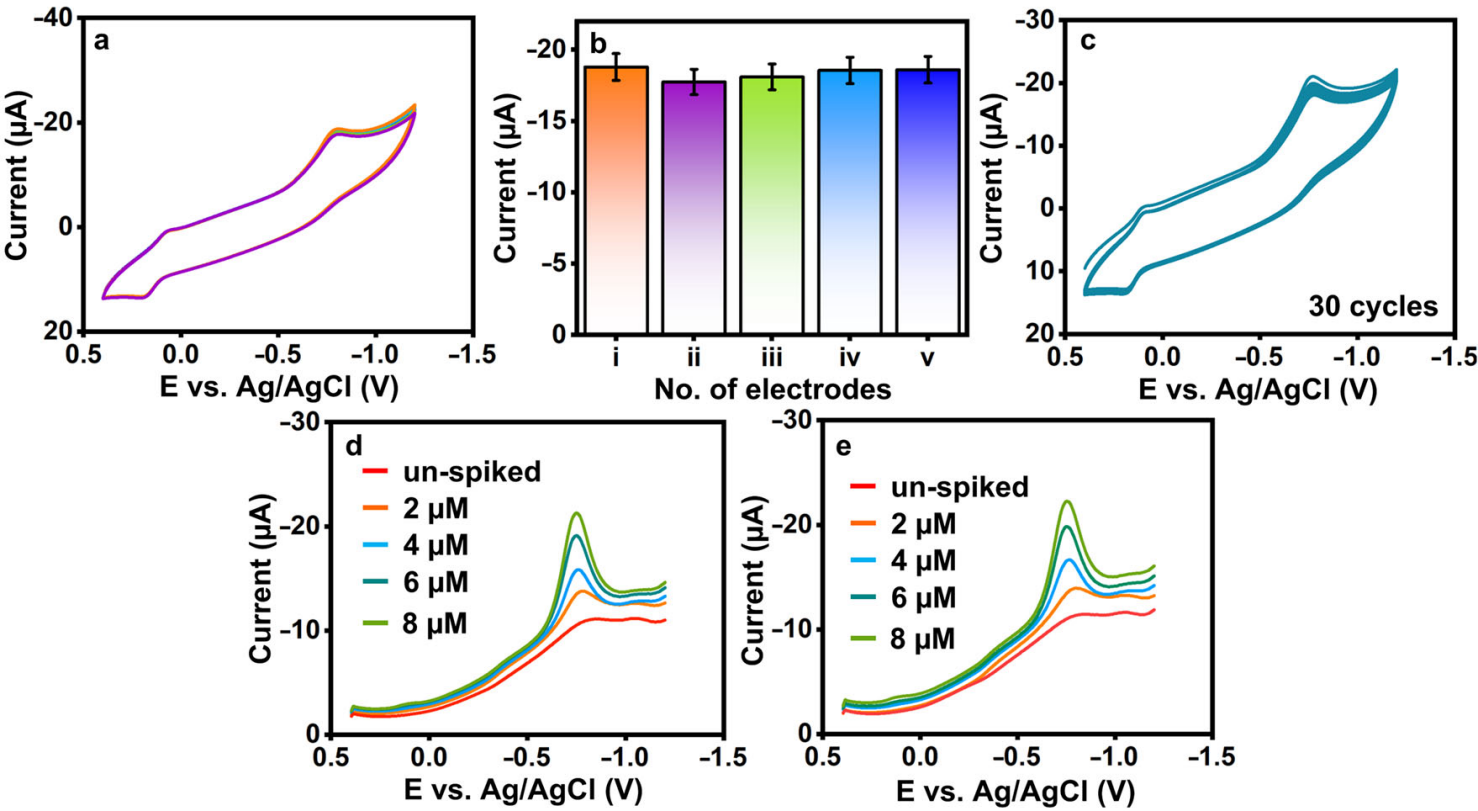
(**a**,**b**) Reproducibility and (**c**) cycle stability of La_2_Sn_2_O_7_/*f*-HNT/SPCE with the presence of 3-nitro-l-tyrosine. (**d**,**e**) Un-spiked and spiked 3-nitro-l-tyrosine samples at La_2_Sn_2_O_7_/*f* -HNT/SPCE.

The reproducibility (Figure 6a,b) and cycle stability (Figure 6c) of 3-nitro-l-tyrosine were evaluated at La_2_Sn_2_O_7_/*f*-HNT/SPCE in 0.1 M pH-7. Five La_2_Sn_2_O_7_/*f*-HNT-modified SPCEs individually examined the fabricated sensor’s reproducibility, as shown in Figure 6a,b. Finally, it was determined that the sensor’s RSD was 3-nitro-l-tyrosine = ±1.89%, which was more tolerable. Additionally, the constructed sensor’s operational cycle stability was tested for 30 cycles with 3-nitro-l-tyrosine, as demonstrated in Figure 6c. The reduction current of 3-nitro-l-tyrosine shows no significant variations from the first cycle to the thirtieth cycle value was estimated to be 3-nitro-l-tyrosine ±4.91%. In conclusion, La_2_Sn_2_O_7_/*f*-HNT/SPCE exhibits acceptable reproducibility and stability for the detection of 3-nitro-l-tyrosine. The shelf-life and interference studies were provided in the supporting information (Figures S1 and S2).

#### 3.2.4. Spiked Sample Analysis

To evaluate the practicality and effectiveness of the sensor, the sensor was tested using real samples of saliva and urine, as depicted in Figure 6d,e, respectively. Each sample category underwent specific preparation and spiking techniques. The measurement of spiked 3-nitro-l-tyrosine in selected models was carried out using the standard addition procedure. Initially, the real samples of saliva and urine showed negligible levels of 3-nitro-l-tyrosine, which prompted the introduction of varied amounts (*n* = 3) of spiked 3-nitro-l-tyrosine under similar conditions. The La_2_Sn_2_O_7_/f-HNT/SPCE sensor demonstrated promising performance, exhibiting good recovery rates ranging from ±96.41% to 98.32% for 3-nitro-l-tyrosine detection in the real samples. These results validate the sensor’s capability to accurately detect and quantify 3-nitro-l-tyrosine in the presence of complex matrices, thus highlighting its potential as a reliable and efficient analytical tool for real sample analysis.

## 4. Conclusions

In this study, we successfully synthesized La_2_Sn_2_O_7_/*f*-HNT nanocomposites using a surfactant-assisted method, which allowed for the controlled and efficient incorporation of La_2_Sn_2_O_7_ nanoparticles onto the surface of *f*-HNT. The resulting nanocomposite was characterized extensively using X-ray diffraction (XRD), Fourier-transform infrared spectroscopy (FT-IR), and transmission electron microscopy (TEM) techniques, confirming the successful formation of the desired composite structure. The XRD analysis revealed the presence of characteristic peaks corresponding to La_2_Sn_2_O_7_ and *f*-HNT, confirming their coexistence within the nanocomposite. The FT-IR analysis provided valuable information about the bonding and functional groups present in the composite, further supporting its composition. TEM images showcased the nanoscale morphology of the La_2_Sn_2_O_7_/*f*-HNT nanocomposite, highlighting the uniform distribution of La_2_Sn_2_O_7_ nanoparticles on the *f*-HNT surface. The synthesized La_2_Sn_2_O_7_/*f*-HNT/SPCE electrode exhibited excellent catalytic properties for the detection of 3-nitro-l-tyrosine. The sensor demonstrated high sensitivity, achieving low detection limits, and a wide linear range for the accurate quantification of 3-nitro-l-tyrosine in saliva and urine samples. These findings indicate the potential of the La_2_Sn_2_O_7_/*f*-HNT/SPCE electrode as a reliable platform for the specific and sensitive detection of 3-nitro-l-tyrosine in real-world samples. The combination of the unique properties of La_2_Sn_2_O_7_ and *f*-HNT, such as their high surface area, enhanced electronic conductivity, and catalytic activity, contributed to the superior performance of the sensor. This novel platform holds promise for the development of advanced sensing technologies and analytical methods for the detection and monitoring of 3-nitro-l-tyrosine in biomedical and environmental applications. Further research and optimization efforts can explore the full potential of the La_2_Sn_2_O_7_/*f*-HNT nanocomposite and expand their application scope in various fields of chemical and biological analysis.

## Data Availability

Data available on request.

## Supplementary Materials

The following supporting information can be downloaded at https://www.mdpi.com/article/10.3390/bios13070722/s1, chemicals and reagents; instrumentation and methods; preparation of thin films for transmission electron microscopy; sample grinding for X-Ray diffraction; pellet preparation for Fourier Transform-Infrared Spectroscopy; interference study; shelf-life stability; Table S1: Evaluation of analytical limits for the determination of NO_2_–Tyr with previous reports.
