# Supplementary material for: The Fabrication of a La2Sn2O7/f-HNT Composite for Non-Enzymatic Electrochemical Detection of 3-Nitro-l-tyrosine in Biological Samples
Source: Biosensors (Basel). 2023 Jul 10;13(7):722. doi: 10.3390/bios13070722 (PMC10377610; doi:10.3390/bios13070722)
Supplement: Supplementary file 1 [file biosensors-13-00722-s001.zip › biosensors-2451532-supplementary.pdf]

Supplementary material

Chemicals and reagents

Ultrapure fresh water is obtained from a millipore water purification system (Milli-Q, specific resistivity > 18 MΩcm, S.A.; Molsheim, France) and is used in all the experiments. Sodium phosphate dibasic and sodium dihydrogen phosphate (Na_2HPO_4 and NaH_2PO_4) are utilized to prepare 0.1 M PB (phosphate buffer) (pH–7). All the electrochemical experiments are carried out using 0.1 M PB (pH–7) as the supporting electrolyte.

Instrumentation and methods

Phase configuration is identified using an X-ray diffraction analysis (XRD) (Bruker, Rigaku D/maxB, DMX-2200)) instrument. The Fourier-transform infrared (FTIR) spectra were recorded by using an FTIR spectrophotometer (JASCO 6600). The surface morphology and the elemental composition are studied utilizing field emission scanning electron microscopy (FE-SEM by Hitachi S-3000H) and high-resolution (HR) transmission electron microscopy (TEM) (H–7600, Hitachi–Japan) operating at 200 kV, and energy dispersive X-ray spectroscopy using JOEL Serive Advanced Technology. By utilizing these characterization methods, the physical properties of the as-prepared materials are investigated. The CHI 1211c electrocatalytic workstation is functional for carrying out electrochemical measurements like cyclic voltammetry (CV) and differential pulse voltammetry (DPV) in a conventional three-electrode cell. Here, the modified SPCE (surface area = 0.072 cm^2), saturated Ag|AgCl, and Pt wire are active as working, reference, and counter electrodes, respectively.

Preparation of thin films for Transmission Electron Microscopy (TEM)

A tiny amount of the nanocomposite sample is dispersed in ethanol or another suitable solvent.

This dispersion is then sonicated for a short duration to ensure the sample is evenly distributed.

A drop of this dispersion is placed on a TEM grid and allowed to air dry before analysis.

Sample grinding for X-Ray Diffraction (XRD)

A sufficient quantity of the nanocomposite is taken and ground to a fine powder.

This ensures a homogenous sample and allows for better interaction with the incident X-rays during the analysis.

The powdered sample is then loaded onto an XRD sample holder and leveled to ensure a smooth, flat surface.

Pellet preparation for Fourier-Transform Infrared Spectroscopy (FT-IR)

The nanocomposite is mixed with spectroscopic grade KBr (potassium bromide) in a suitable ratio, typically around 1:100.

This mixture is then ground to a fine, homogenous powder.

The powder is placed in an evacuable pellet die and subjected to high pressure to form a transparent pellet.

This pellet is then ready for FT-IR analysis.

Interference study

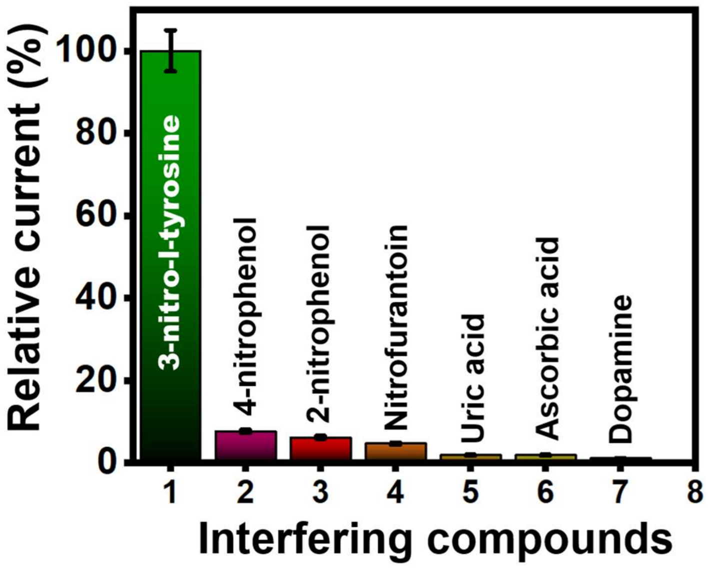

Figure S1. Anti-interference ability of the $La_2Sn_2O_7$/*f*-HNT-modified electrode with the presence of 3-nitro-l-tyrosine and the co-existence of an excess concentration (20–fold) of co-interfering compounds.

Shelf-life Stability

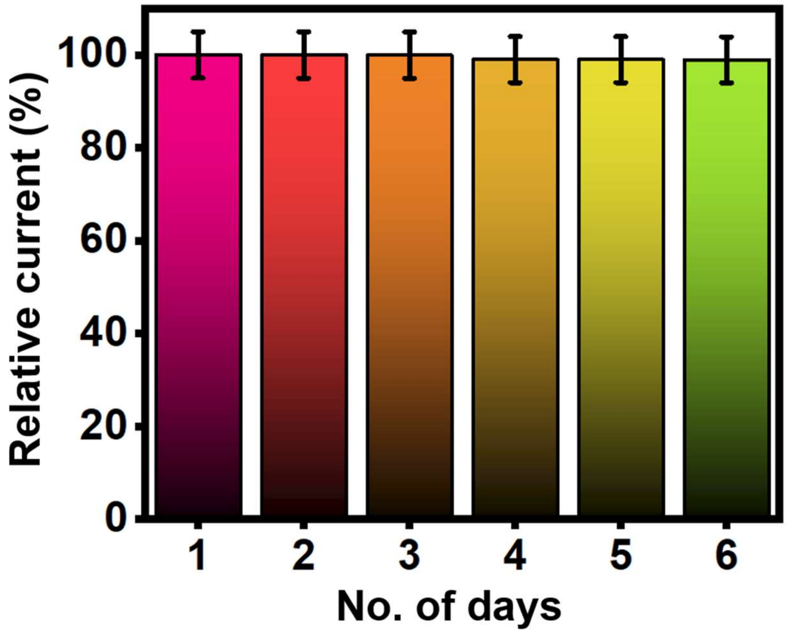

Figure S2. The shelf-life stability study was conducted to assess the endurance and practical application of our fabricated electrodes. We created six $La_2Sn_2O_7$/*f*-HNT-modified electrodes using the established fabrication process and stored them under controlled conditions for six days. Each day, the electrodes were thoroughly tested to detect any potential fluctuation in their performance, providing critical insights into their stability over time. Remarkably, even after six days, the electrodes displayed an impressive 96% of their initial electrochemical response, demonstrating their robustness and durability. These findings suggest that the sensors have a substantial shelf-life and are suitable for long-term use, which is crucial for real-world applications where consistent and reliable performance is required.

Table S1. Evaluation of the analytical limits for the determination of 3-nitro-l-tyrosine with previous reports.

Electrodes	Methods	Linear ranges (µM)	LOD (nM)	Ref.
ZrO_2@rGO	*i-t*	0.025–855.2	9	S1
$ZnNb_2O_6$/*f*-CNF	DPV	0.25–277.5	21	S2
BMIP@CDs	Fluorescence	0.050–1.85	17	S3
AuNPs/MIP/CNT@GO	SWSV	0.2–50.0	50	S4
**$La_2Sn_2O_7$/*f*-HNT**	**DPV**	**0.5–214**	**12**	**This work**

References

S1. Maheshwaran, S.; Akilarasan, M.; Chen, S.-M.; Chen, T.-W.; Tamilalagan, E.; Tzu, C. Y.; Lou, B.-S., An ultra-sensitive electrochemical sensor for the detection of oxidative stress biomarker 3-nitro-l-tyrosine in human blood serum and saliva samples based on reduced graphene oxide entrapped zirconium (IV) oxide. Journal of the Electrochemical Society 2020, 167 (6), 066517.

S2. Priscillal, I. J, Wang, S. F. Coral reef-like zinc niobate nanostructures decorated functionalized carbon nanofiber as electrode modifier for detection of oxidative stress biomarker: 3-nitro-L-tyrosine. Materials Today Chemistry 2022, 1;25:100970.

S3. Jalili, R.; Amjadi, M., Bio-inspired molecularly imprinted polymer–green emitting carbon dot composite for selective and sensitive detection of 3-nitrotyrosine as a biomarker. Sensors and Actuators B: Chemical 2018, 255, 1072-1078.

S4. Wang, S., Sun, G., Chen, Z., Liang, Y., Zhou, Q., Pan, Y. and Zhai, H., 2018. Constructing a novel composite of molecularly imprinted polymer-coated AuNPs electrochemical sensor for the determination of 3-nitrotyrosine. Electrochimica Acta, 259, pp.893-902.
